# ETV4 promotes breast cancer cell stemness by activating glycolysis and CXCR4-mediated sonic Hedgehog signaling

**DOI:** 10.1038/s41420-021-00508-x

**Published:** 2021-05-29

**Authors:** Tao Zhu, Juyan Zheng, Wei Zhuo, Pinhua Pan, Min Li, Wei Zhang, Honghao Zhou, Yang Gao, Xi Li, Zhaoqian Liu

**Affiliations:** 1 Departments of Clinical Pharmacology and Respiratory Medicine, Hunan Key Laboratory of Pharmacogenetics, and National Clinical Research Center for Geriatric Disorders, Xiangya Hospital, Central South University, Changsha, China; 2 Institute of Clinical Pharmacology, Engineering Research Center for applied Technology of Pharmacogenomics of Ministry of Education, Central South University, Changsha, China; 3 Department of Thoracic Surgery, Xiangya Hospital, Central South University, Changsha, China

**Keywords:** Cancer metabolism, Cancer stem cells

## Abstract

Cancer stem cells (CSCs) are a major cause of tumor treatment resistance, relapse and metastasis. Cancer cells exhibit reprogrammed metabolism characterized by aerobic glycolysis, which is also critical for sustaining cancer stemness. However, regulation of cancer cell metabolism rewiring and stemness is not completely understood. Here, we report that ETV4 is a key transcription factor in regulating glycolytic gene expression. ETV4 loss significantly inhibits the expression of HK2, LDHA as well as other glycolytic enzymes, reduces glucose uptake and lactate release in breast cancer cells. In human breast cancer and hepatocellular carcinoma tissues, ETV4 expression is positively correlated with glycolytic signaling. Moreover, we confirm that breast CSCs (BCSCs) are glycolysis-dependent and show that ETV4 is required for BCSC maintenance. ETV4 is enriched in BCSCs, its knockdown and overexpression suppresses and promotes breast cancer cell stem-like traits, respectively. Mechanistically, on the one hand, we find that ETV4 may enhance glycolysis activity to facilitate breast cancer stemness; on the other, ETV4 activates Sonic Hedgehog signaling by transcriptionally promoting CXCR4 expression. A xenograft assay validates the tumor growth-impeding effect and inhibition of CXCR4/SHH/GLI1 signaling cascade after ETV4 depletion. Together, our study highlights the potential roles of ETV4 in promoting cancer cell glycolytic shift and BCSC maintenance and reveals the molecular basis.

## Introduction

Breast cancer is the most commonly occurring cancer and remains the leading cause of cancer-related deaths in women worldwide^1,2^. Although a reduction in mortality rate has been achieved owing to early detection and improved therapeutic regimens, drug resistance, relapse and metastasis still severely threaten the survival of breast cancer patients^3–7^. Cancer stem cells (CSCs), a subpopulation of cells with self-renewal ability and multi-lineage differentiation potential, play a dominant role in tumor initiation and propagation^8–10^. Since the demonstration of CSCs in breast cancer, accumulating evidence indicates that these breast cancer stem cells (BCSCs) lie behind refractory, recurrent and metastatic lesions. ALDH1-positive BCSCs in breast cancer patients were associated with low response to paclitaxel and epirubicin-based chemotherapy^11^. Relapse of breast cancer due to the exitance of treatment-resistant CSCs has a strong correlation with metastatic disease. In a murine metastatic model, only the BCSCs population, but not differentiated breast cancer cells, can migrate and grow at distant sites^12^. Hence, elimination of BCSCs is crucial for successful breast cancer treatment, which requires a comprehensive understanding of BCSCs. However, it remains incompletely clarified how the key properties of BCSCs are regulated.

Metabolic reprogramming is a hallmark of cancer, characterized by a shift toward glycolysis even in the presence of oxygen (Warburg effect)^13–15^. A glycolytic phenotype endues cancer cells with building blocks required for rapid proliferation, and the end product lactate can not only serve as an energy source for neighboring cancer cells^16^, but also acidify the tumor microenvironment (TME), an acidic TME is conducive for cancer progression because it promotes the metastasis of cancer cells and suppresses the function of immune cells^15,17^. Glycolysis involves a series of chemical reactions catalyzed by specific enzymes, many of which are overexpressed in cancer tissues and correlate with poor prognosis, such as HK2^18,19^, PGK1^20,21^, PKM2^22,23^, LDHA^24^. Regulation of glycolytic enzymes is an efficient way to orchestrate cellular glycolysis. HIF-1α, c-Myc, and p53 are well-established glycolysis-associated transcription factors by directly activating or suppressing transcription of glycolytic genes^14^. More recently, SIX1 was identified as a key transcription factor promoting glycolytic enzymes expression^25^. Despite that several transcription factors involved in glycolysis regulation have been identified, transcriptional regulation of this metabolic shift needs further elucidation.

ETV4 belongs to the E-twenty-six (ETS) transcription factor superfamily. Like other members in this family, ETV4 is characterized by the ETS DNA-binding domain which recognizes a GGAA/T core consensus motif^26,27^. By activating transcription of its target genes, ETV4 plays relevant roles in biological processes, including embryogenesis^26^, hippocampal and kidney development^28,29^. Upregulated expression and a pro-tumoral effect of ETV4 have also been revealed in multiple malignancies^30–34^. For example, increasing evidence shows that ETV4 is able to promote breast cancer metastasis by transcriptionally activating expression of epithelial-mesenchymal transition (EMT) inducers such as ZEB1 and SNAIL1^35,36^, and of extracellular matrix-degrading proteinases such as MMP2 and MMP9^37^. To date, little is known about the roles of ETV4 in cancer cell energy metabolism and maintenance of cancer stemness.

A previous study indicated that ETV4 was capable of forming a complex with HIF-1α to regulate hypoxic gene expression^38^, which inspired us to wonder whether ETV4 is involved in glycolytic shift. Besides, we also queried whether ETV4 contributes to CSC properties considering the energy requirements of CSCs. In this study, we report that ETV4 is a critical transcription factor for metabolic rewiring in cancer cells, it promotes the transcription of hexokinase 2 (HK2) and lactate dehydrogenase A (LDHA), two enzymes indispensable for efficient glycolytic flux. Knockdown of ETV4 inhibits glycolysis activity. ETV4 is also required for stem-like traits in breast cancer cells. Mechanistically, on the one hand, breast cancer cell stemness relies on glycolytic metabolism which may be regulated by ETV4. On the other hand, ETV4 activates the sonic Hedgehog signaling, one of the signaling pathways closely associated with cancer stemness, via transcriptionally upregulating the chemokine receptor CXCR4. These findings not only indicate a novel role of ETV4 in regulating cellular glycolysis by transcriptionally augmenting the expression of glycolytic enzymes, but also reveal the effect of ETV4 in promoting cancer stemness via two distinct mechanisms. Our study highlights the potential of ETV4 as a promising target to simultaneously suppress glycolysis and cancer cell stemness in breast cancer.

## Results

### ETV4 promotes expression of HK2 and LDHA in breast cancer cells

**Fig. 1:**
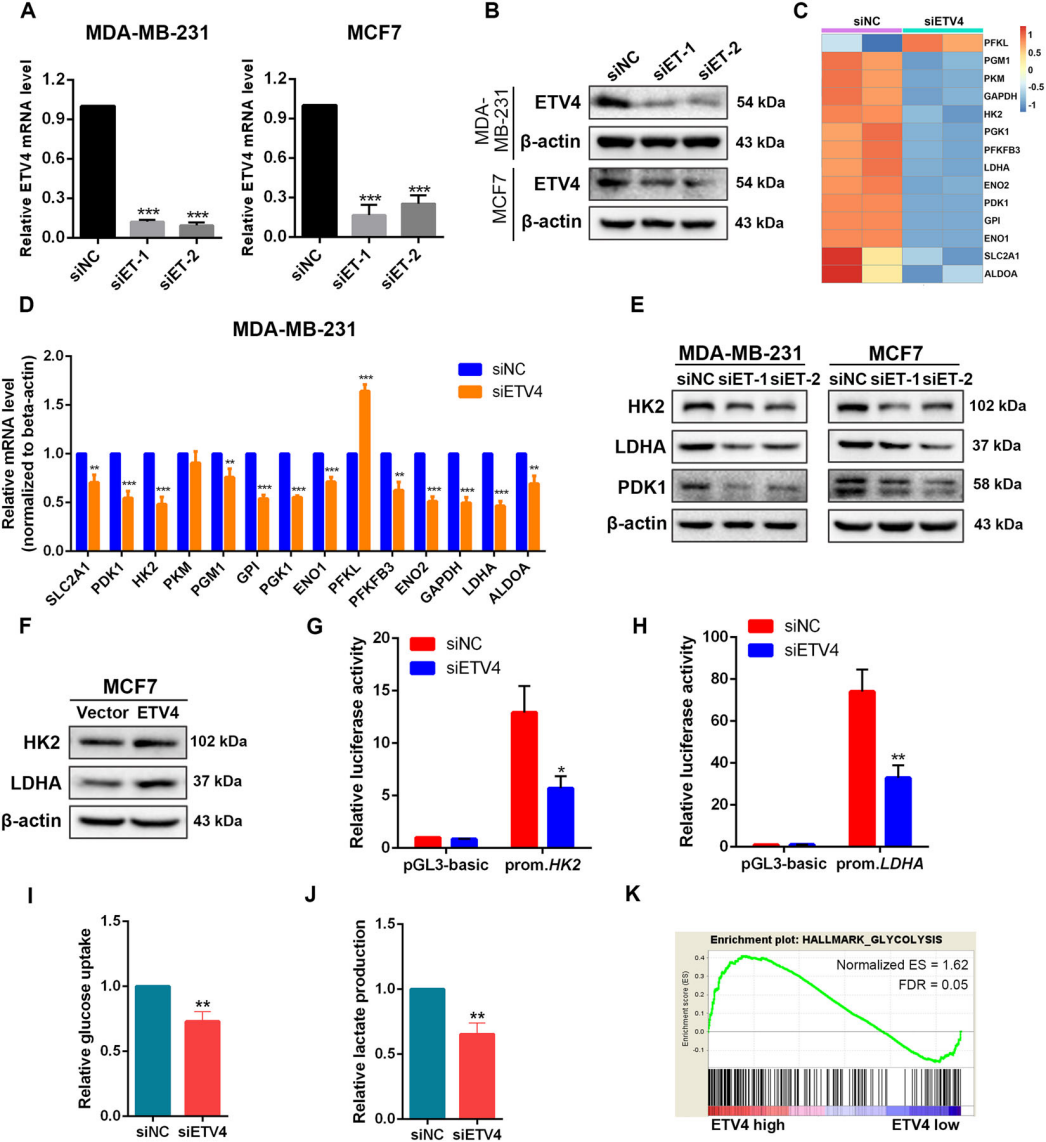
Depletion of ETV4 suppresses glycolysis by inhibiting HK2 and LDHA expression in breast cancer cells. **A**, **B** ETV4 expression at the mRNA (**A**) and protein (**B**) levels in MDA-MB-231 and MCF7 breast cancer cells transfected with siRNA targeting ETV4 (siET) or negative control siRNA (siNC). **C** Heatmap displaying glycolytic gene expression measured by RNA sequencing in ETV4-silenced MDA-MB-231 cells. **D** Quantitative RT‐PCR measurement of glycolytic gene expression in MDA-MB-231 cells transfected with siET or siNC. **E** Immunoblots of HK2, LDHA, and PDK1 in control or ETV4-silenced MDA-MB-231 and MCF7 cells. **F** Immunoblots of HK2 and LDHA in control and ETV4-overexpressing MCF7 cells. **G**, **H** Luciferase activity of *HK2* promoter reporter (**G**) and *LDHA* promoter reporter (**H**) in MDA-MB-231 cells with or without ETV4 knockdown. prom, promoter (**I** and **J**) Relative glucose uptake (**G**) and relative lactate production (**H**) in control or ETV4-silenced MDA-MB-231 cells. **K** GSEA plot showing that ETV4 expression was significantly correlated with glycolysis activity in TCGA breast cancer dataset. Data were presented as mean ± SD, *n* = 3. **P < 0.01 and ***P < 0.001.

Since ETV4 is a coactivator of hypoxia-inducible factor signaling^38^, we hypothesized that ETV4 could serve as a regulator of glycolytic metabolism to regulate cancer cell growth. To test this hypothesis, we first measured the expression of glucose transporters and key glycolytic enzymes after knockdown of ETV4 in two human hepatocellular carcinoma cell lines HepG2 and Huh7, which show high glycolytic activities^39^. A significant reduction in the mRNA expression of SLC2A1, SLC2A4, GPI, ALDOA, GAPDH, ENO1, PKM2 and LDHA, but not HK2, was observed in these two cell lines after ETV4 expression was silenced (Supplementary Fig. 1). To confirm these results in human breast cancer cells, ETV4 was knocked down by specific siRNAs in MDA-MB-231 and MCF-7 cells (Fig. 1A and B). We performed RNA sequencing analysis in ETV4-silenced and control MDA-MB-231 cells and found downregulation of a variety of glycolytic enzymes except PFKL after ETV4 knockdown (Fig. 1C). Similar results were observed by real-time quantitative PCR analysis (Fig. 1D). These findings reveal a broad effect of ETV4 in regulating expression of glycolytic genes at the transcriptional level. Notably, the effects of ETV4 knockdown on the mRNA expression of HK2 and PKM2 are different in HCC and breast cancer cells (Supplementary Fig. 1, Fig. 1C), suggesting a cancer type-specific regulation of these two enzymes by ETV4. ETV4 knockdown also downregulated HK2, LDHA and PDK1 protein levels in breast cancer cells (Fig. 1E). Since the baseline expression level of ETV4 in MCF7 cells was lower than that of MDA-MB-231 cells, we overexpressed ETV4 in MCF7 cells (Supplementary Fig. 2). Consistently, forced expression of ETV4 upregulated HK2 and LDHA protein expression (Fig. 1F). We next performed dual luciferase assays to ask whether ETV4 regulates HK2 and LDHA expression by activating their transcription. Promoter reporters containing the putative promoter regions, 2 kb of sequence upstream of the transcription start site, of *HK2* and *LDHA* were constructed. As shown in Fig. 1G, H, ETV4 knockdown decreased the activity of *HK2* and *LDHA* promoter reporters, which provide evidence that ETV4 transcriptionally activates glycolytic gene expression. Furthermore, loss of ETV4 rendered MDA-MB-231 cells an impaired ability to uptake glucose, and a decreased level of lactate in the medium was also observed (Fig. 1I, J). To investigate the glycolytic effect of ETV4 expression in cancer tissues, we performed gene set enrichment analysis (GSEA) by exploiting The Cancer Genome Atlas (TCGA) RNA-sequencing data. The GSEA results revealed that ETV4 expression was significantly correlated with glycolysis activity in breast cancer and HCC tissues (Fig. 1K and Supplementary Fig. 1).

In light of the function of glycolysis to support synthesis of macromolecules essential for cell proliferation, it is reasonable to expect that downregulation of ETV4 to disrupt glycolytic flow will hinder cancer cell growth. Consistently, we found that ETV4 was upregulated in breast cancer tissues according to analysis of GSE42568 dataset, and we confirmed ETV4 upregulation in the majority of cancer tissues in an independent breast cancer cohort, with 8 of 12 tumors expressing significantly higher ETV4 mRNA levels (Supplementary Fig. 3). Knockdown of ETV4 rendered MDA-MB-231 and MCF7 cells impaired proliferation (Supplementary Fig. 3), which is in line with previous research^30^. Moreover, high ETV4 levels were significantly associated with shorter distant relapse free survival (Supplementary Fig. 3).

### ETV4 is required for breast cancer stem-like traits

**Fig. 2:**
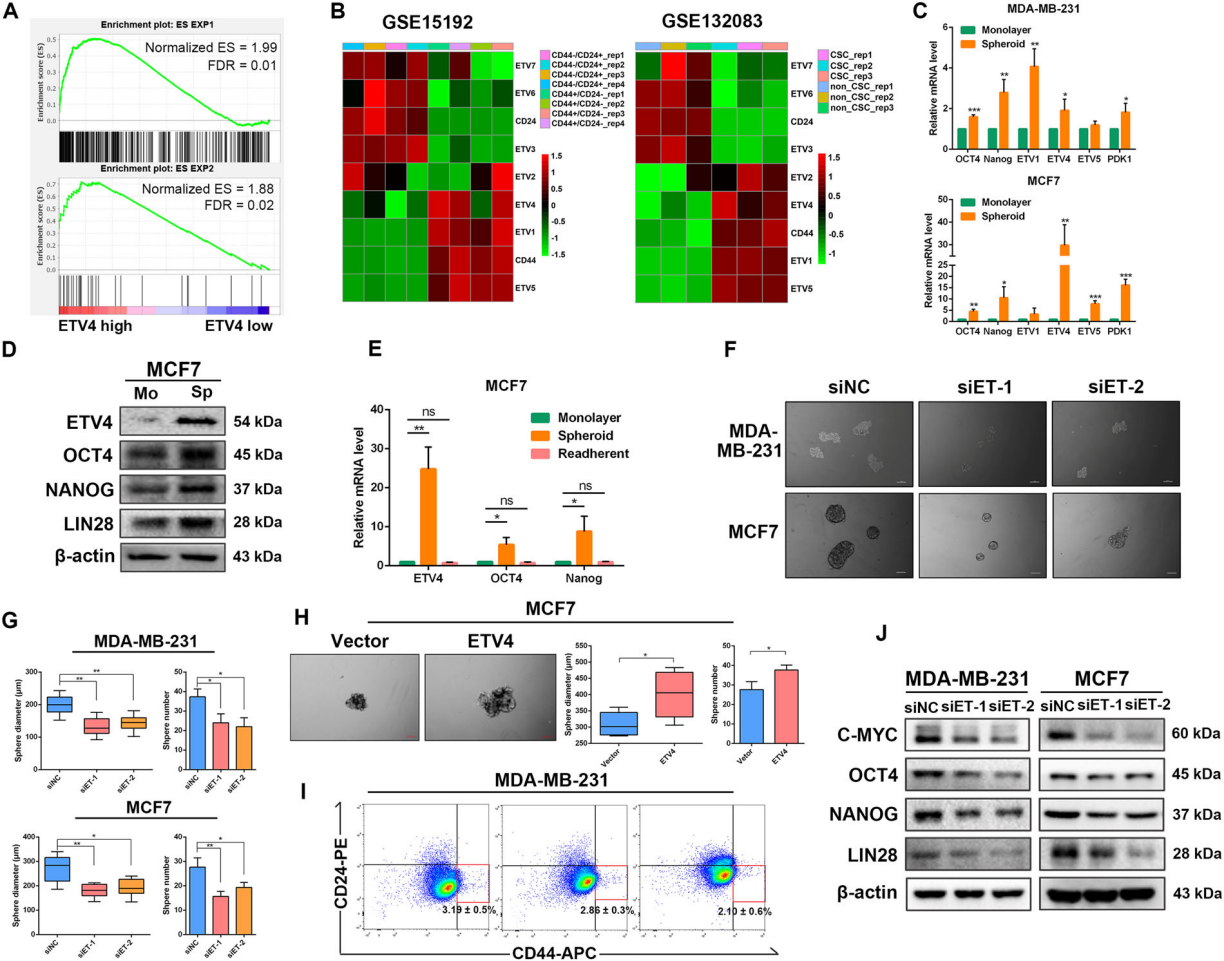
ETV4 is required for breast cancer cell stem-like characteristics. **A** GSEA plot displaying that the ES1 target genes were strongly enriched in TCGA breast cancer tissues with high ETV4 expression. **B** Heatmap of the expression signature of PEA3 subfamily members between stem cell and non-stem cell subpopulations of MCF10A and SUM149 cells using gene expression data from GSE15192 and GSE132083. **C** Quantitative RT‐PCR measurement of cancer stem cell markers (OCT4 and NANOG) and PEA3 subfamily members (ETV1, ETV4 and ETV5) in monolayer MDA-MB-231 cells and their spheres, monolayer MCF7 cells and their spheres. **D** Immunoblots of ETV4 and cancer stem cell markers (OCT4, NANOG and LIN28) in monolayer MCF7 cells and MCF7-derived spheres. **E** Quantitative RT‐PCR measurement of ETV4, OCT4 and NANOG in MCF7 spheres and re-adherent MCF7 cells. **F**, **G** Representative images of spheres formed by the indicated cells with or without ETV4 depletion (**F**) and histograms showing the diameters and numbers of spheres (**G**). Scale bar =100 μm. **H** Representative images of spheres of MCF7 cells with or without ETV4 overexpression (left panel) and boxplots or histograms showing the diameters and numbers of spheres (right panel). A box represents the distribution of sphere diameter and a upper (lower) whisker represents the max (min) diameter. Scale bar =100 μm. **I** The subpopulation of CD44^+^/CD24^-^ cells in control and ETV4-silenced MDA-MB-231 cells as assessed by flow cytometry. **J** Immunoblots of C-MYC, OCT4, NANOG and LIN28 in MDA-MB-231 and MCF7 cells with or without ETV4 knockdown. Data are presented as mean ± SD, *n* = 3. *P < 0.05, **P < 0.01, ***P < 0.001.

Considering that CSCs can be highly glycolytic or oxidative phosphorylation (OXPHOS) dependent, and that ETV4 is specifically expressed in undifferentiated embryonic stem cells^40,41^, we next explored whether ETV4 was involved in stem-like properties in breast cancer cells. By performing GSEA of TCGA breast cancer dataset, we found that stem-associated gene signaling (two different gene sets associated with human embryonic stem cell identity^42^) was markedly enriched in the high ETV4 expression group (Fig. 2A). We next compared ETV4 expression between stem cell subpopulations and non-stem cell subpopulations of human breast epithelial MCF10A cells and breast cancer SUM149 cells using two public datasets GSE15192^43^ and GSE132083. In the stem cell subpopulations, the mRNA levels of ETV4 and the other two members of the PEA3 family, ETV1 and ETV5, were significantly increased (Fig. 2B). Consistently, real-time quantitative PCR revealed that the mRNA levels of ETV4 in BCSCs-enriched spheres, as evidenced by upregulated expression of stemness-related factors including OCT4, NANOG, were significantly higher than those in the corresponding adherent MDA-MB-231 and MCF7 counterparts (Fig. 2C). Immunoblotting assays in MCF7 cells confirmed elevated protein expression of ETV4 and stemness-related factors in BCSCs-enriched spheres (Fig. 2D). When the spheroid cells were digested into single cells and re-attached, the elevated expression of ETV4, OCT4 and NANOG returned to levels comparable to those in untreated adherent cells (Fig. 2E). Upregulation of ETV4 in BCSCs prompted us to speculate that ETV4 is necessary for sustaining breast cancer stemness. We then investigated the effect of ETV4 on stem-like properties by manipulating ETV4 expression. Knockdown of ETV4 in MCF7 and MDA-MB-231 cells remarkably inhibited their sphere formation abilities, as the diameters and numbers of spheres were significantly reduced in ETV4-depleted cells compared with control cells (Fig. 2F, G), whereas ETV4-overexpressing MCF7 cells showed an increased sphere formation ability (Fig. 2H). Moreover, ETV4-silenced MDA-MB-231 cells exhibited a significant decrease in the percentage of CD44^+^/CD24^−^ subpopulation as determined by flow cytometric analysis (Fig. 2I). Loss of ETV4 markedly downregulated the protein levels of stemness-related factors (Fig. 2J). Taken together, these results demonstrate that ETV4 is required for CSC-like properties in breast cancer cells.

### Blockage of glycolysis reverses ETV4 overexpression-induced stem-like traits

**Fig. 3:**
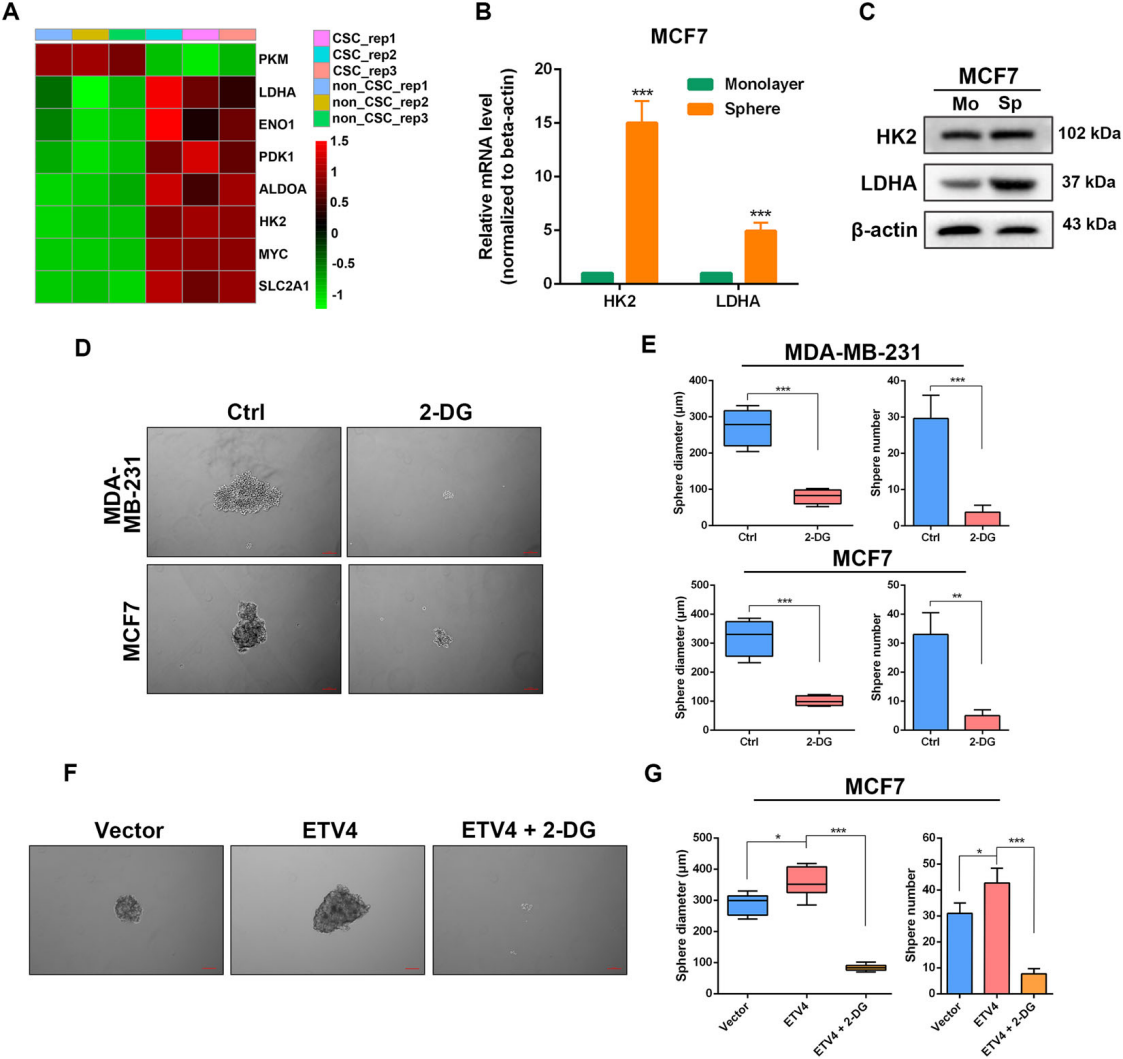
Inhibition of glycolysis reverses ETV4 overexpression-induced stem-like properties in breast cancer cells. **A** Heatmap showing glycolytic gene expression between CSC and non-CSC subpopulations using transcriptome sequencing data of SUM149 breast cancer cells from GSE132083. **B**, **C** Quantitative RT‐PCR measurement of HK2 and LDHA (**B**), immunoblots of HK2 and LDHA (**C**) in monolayer MCF7 cells and MCF7 spheres. **D**, **E** Representative images of spheres formed by the indicated cells treated with or without 20 mM 2-DG (**D**) and boxplots or histograms showing the diameters and numbers of spheres (**E**). **F**, **G** Representative images of spheres formed by ETV4-overexpressing MCF7 cells treated with or without 20 mM 2-DG (**F**) and boxplots or histograms showing the diameters and numbers of spheres (**G**). A box represents the distribution of sphere diameter and a upper (lower) whisker represents the max (min) diameter. Data are presented as mean ± SD, *n* = 3. ns, not significant, *P < 0.05, **P < 0.01, ***P < 0.001.

We next asked whether ETV4 promotes breast cancer cell stem-like properties by regulating glycolysis. Using transcriptome sequencing data of the CSC subpopulation in SUM149 human breast cancer cell line from GSE132083, we compared the glycolytic gene expression profiles between the CSC group and non-CSC group, and found that SLC2A1, HK2, ALDOA, ENO1, LDHA and PDK1 were upregulated in the CSC group (Fig. 3A). Further confirming these results, the mRNA and protein levels of HK2 and LDHA were significantly higher in CSC-enriched spheres than those in the corresponding monolayer MCF7 cells, as detected by real-time quantitative PCR and western blotting (Fig. 3B, C). Next, we used 2-deoxy-D-glucose (2-DG), a glycolytic inhibitor, to block glycolysis in MDA-MB-231 and MCF7 cells and observed that 2-DG treatment remarkably suppressed the sphere formation efficiency in both cell lines (Fig. 3D, E). These results indicate that active glycolytic flux is critical for maintenance of breast cancer cell stemness, which is consistent with a previous study^44^. Moreover, the increased sphere formation ability induced by ETV4 overexpression was reversed by 2-DG treatment in MCF7 cells (Fig. 3F, G). Collectively, these data suggest that ETV4 facilitates maintenance of cancer stemness through regulating glycolysis in breast cancer.

### ETV4 promotes breast cancer cell stemness by activating the sonic HH signaling

**Fig. 4:**
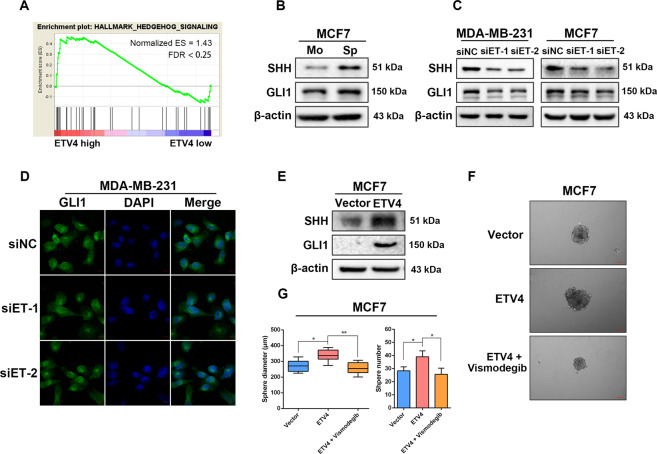
ETV4 promotes breast cancer cell stemness by activating the Sonic HH signaling. **A** GSEA plot showing significant enrichment of hedgehog signaling in TCGA breast cancer tissues with high ETV4 expression. **B**, **C** Immunoblots of SHH and GLI1 in monolayer MCF7 cells and MCF7-derived spheres (**B**) and in MDA-MB-231 and MCF7 cells with or without ETV4 depletion (**C**). **D** Representative confocal images for GLI1 (green) in MDA-MB-231 cells with or without ETV4 knockdown. Scale bar =10 μm. **E** Immunoblots of SHH and GLI1 in the indicated breast cancer cells with or without ETV4 overexpression. **F**, **G** Representative images of spheres formed by ETV4-overexpressing MCF7 cells treated with or without 10 μM Vismodegib (**F**) and boxplots or histograms showing the diameters and numbers of spheres (**G**). A box represents the distribution of sphere diameter and a upper (lower) whisker represents the max (min) diameter. Data are presented as mean ± SD, *n* = 3. *P < 0.05, **P < 0.01.

Since CSCs rely on a variety of embryonic signaling pathways to fulfill their functions^45^, and ETV4 plays a role in regulating embryogenesis^26^, we explored whether ETV4 could contribute to maintaining cancer stemness by modulating an embryonic signaling pathway. GSEA of the TCGA breast cancer dataset revealed that ETV4 expression was positively correlated with the Hedgehog signaling (Fig. 4A), one of the embryonic development-related pathways implicated in stem-cell maintenance^45,46^. In canonical Hedgehog signaling, the sonic Hedgehog (SHH) ligand binds to its transmembrane receptor Patched (PTCH1) to derepress smoothened (SMO), another transmembrane protein, leading to activation of downstream intracellular effectors, the GLI family of zinc-finger transcription factors (GLI1, GLI2 and GLI3)^47^. Once activated, the GLI proteins translocate to the nucleus to regulate the transcription of their target genes such as NANOG and OCT4^48^. Of the three GLI proteins, GLI1 serves to activate Hedgehog target genes. By performing sphere formation assay to enrich CSC-like subpopulation in MCF7 cells, we found that the protein levels of SHH and GLI1 were upregulated in MCF7 spheres (Fig. 4B), which is in line with the understanding that activated SHH signaling is important for BCSC maintenance. To evaluate whether ETV4 stimulate SHH signaling activation, we knocked down ETV4 expression and found decreased protein levels of SHH and GLI1 in ETV4-silenced breast cancer cells (Fig. 4C). ETV4 depletion-induced GLI1 downregulation was further confirmed by the immunofluorescence assay, as the green signal reflecting GLI1 expression was significantly weaker in the ETV4 knockdown cells (Fig. 4D). In agreement, forced expression of ETV4 led to increased expression of proteins of SHH signaling (Fig. 4E). These findings demonstrate a positive regulatory role of ETV4 expression in SHH signaling activation. We next explored the effect of SHH signaling inhibition on ETV4-participated BCSC maintenance by using the SHH pathway inhibitor Vismodegib. As expected, treatment with Vismodegib abolished the enhanced sphere formation ability induced by ETV4 overexpression in MCF7 cells (Fig. 4F, G). Together, these data suggest that ETV4 regulate breast cancer cell stem-like traits by modulating SHH-GLI1 signaling pathway.

### ETV4-induced SHH activation is mediated by CXCR4

**Fig. 5:**
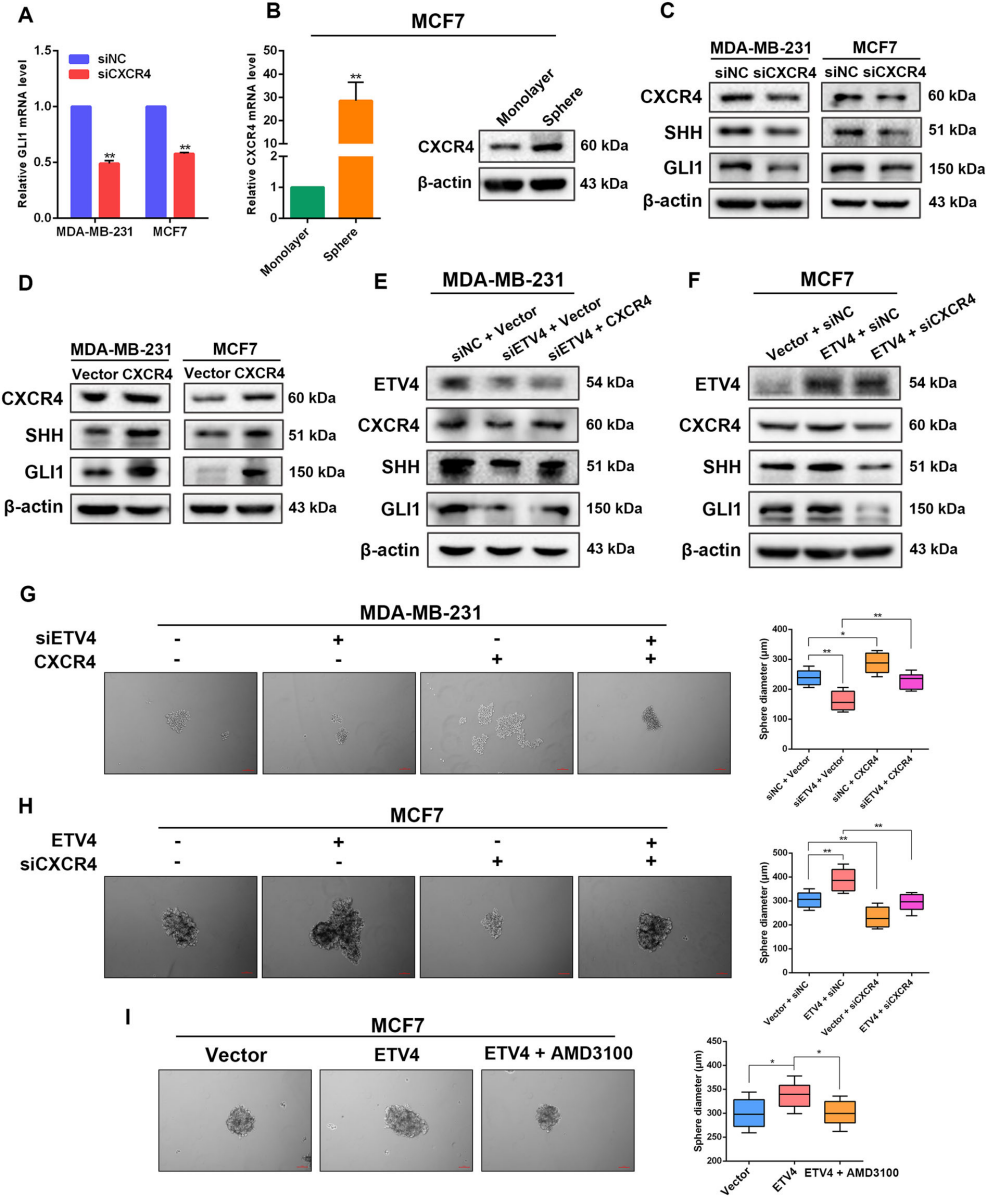
ETV4-induced Sonic HH activation and stem-like traits is mediated by CXCR4. **A** Quantitative RT‐PCR measurement of GLI1 expression in the indicated cells with or without CXCR4 ablation. **B** CXCR4 expression in monolayer MCF7 cells and MCF7-derived spheres. **C**, **D** Immunoblots of CXCR4, SHH and GLI1 in CXCR4-depleted (**C**) and CXCR4-overexpressing (**D**) MDA-MB-231 and MCF7 cells. **E**, **F** Immunoblots of ETV4, CXCR4, SHH and GLI1 in ETV4-silenced MDA-MB-231 cells with or without CXCR4 overexpression (**E**) and in ETV4-overexpressing MCF7 cells with or without CXCR4 depletion (**F**). **G**, **H** Representative images of spheres and histograms showing the sphere diameters formed by control or ETV4-silenced MDA-MB-231 cells with or without CXCR4 overexpression (**G**), and by control or ETV4-overexpressing MCF7 cells with or without CXCR4 knockdown (**H**). **I** Representative images of spheres of ETV4-overexpressing MCF7 cells treated with or without 20 μM AMD3100 and boxplots showing the sphere diameters. A box represents the distribution of sphere diameter and a upper (lower) whisker represents the max (min) diameter. Data are presented as mean ± SD, *n* = 3. *P < 0.05, **P < 0.01.

Next, we investigated the molecular mechanism underlying ETV4-mediated activation of SHH signaling pathway. A previous study has shown that ETV4 promotes the expression of CXCR4, a chemokine receptor expressed by most CSCs, by activating its transcription^49^. In addition, chemokine-based signaling is related to hedgehog pathway activation^50^. Singh et al. reported that in pancreatic cancer cells CXCL12/CXCR4 signaling axis induces sonic hedgehog expression^51^. Based on these findings, it is rational to hypothesize that ETV4 facilitate SHH signaling activation by modulating CXCR4 expression in breast cancer. We then examined the levels of CXCR4 in ETV4-dificient and -overexpressing breast cancer cells. Knockdown of ETV4 clearly reduced the mRNA and protein levels of CXCR4, whereas ETV4 overexpression elevated CXCR4 expression in MDA-MB-231 and MCF7 cells (Supplementary Fig. 4), which confirmed the previous finding that ETV4 activates CXCR4 transcription^49^. To probe the role of CXCR4 in regulating SHH signaling, we first silenced CXCR4 expression and found that CXCR4 depletion led to significantly downregulated mRNA level of GLI1, the effector of hedgehog signaling (Fig. 5A). Moreover, we found elevated expression of CXCR4 in SHH signaling activated breast cancer spheres (Figs. 5B, 4B), indicating that CXCR4 expression is correlated with the activity of SHH pathway. In CXCR4-depleted MDA-MB-231 and MCF7 cells, the protein levels of SHH and GLI1 were decreased as compared with control cells, while ectopic expression of CXCR4 in these cell lines strongly upregulated SHH and GLI1 expression (Fig. 5C, D). These results reveal that CXCR4 plays a role in activating the SHH-GLI1 signaling pathway.

To further demonstrate that CXCR4 is a mediator of the effect of ETV4 on stimulating SHH/GLI1 axis, we examined whether CXCR4 overexpression and knockdown could counteract the effects caused by ETV4 silencing and overexpression, respectively. When CXCR4 was overexpressed in ETV4-silenced MDA-MB-231 cells, suppressed expression of SHH and GLI1 was restored (Fig. 5E). In agreement, in MCF7 cells with relatively low endogenous expression of ETV4, depletion of CXCR4 reversed increases in protein levels of SHH and GLI1 induced by forced expression of exogenous ETV4 (Fig. 5F). Supporting the results of western blotting, we observed that knockdown of CXCR4 clearly abrogated ETV4 overexpression-induced enhancement in sphere formation ability in MCF7 cells, whereas overexpression of CXCR4 rescued the impeded sphere formation efficiency caused by ETV4 deficiency in MDA-MB-231 cells (Fig. 5G, H). Similarly, in the presence of AMD3100, a CXCR4 antagonist, ectopic expression of ETV4 was no longer able to facilitate mammosphere formation (Fig. 5I). Taken together, these results reveal that CXCR4, transcriptionally regulated by ETV4, is a mediator in ETV4-induced SHH pathway activation and stem-like traits in breast cancer cells.

### ETV4 depletion suppressed growth of breast tumor xenograft

**Fig. 6:**
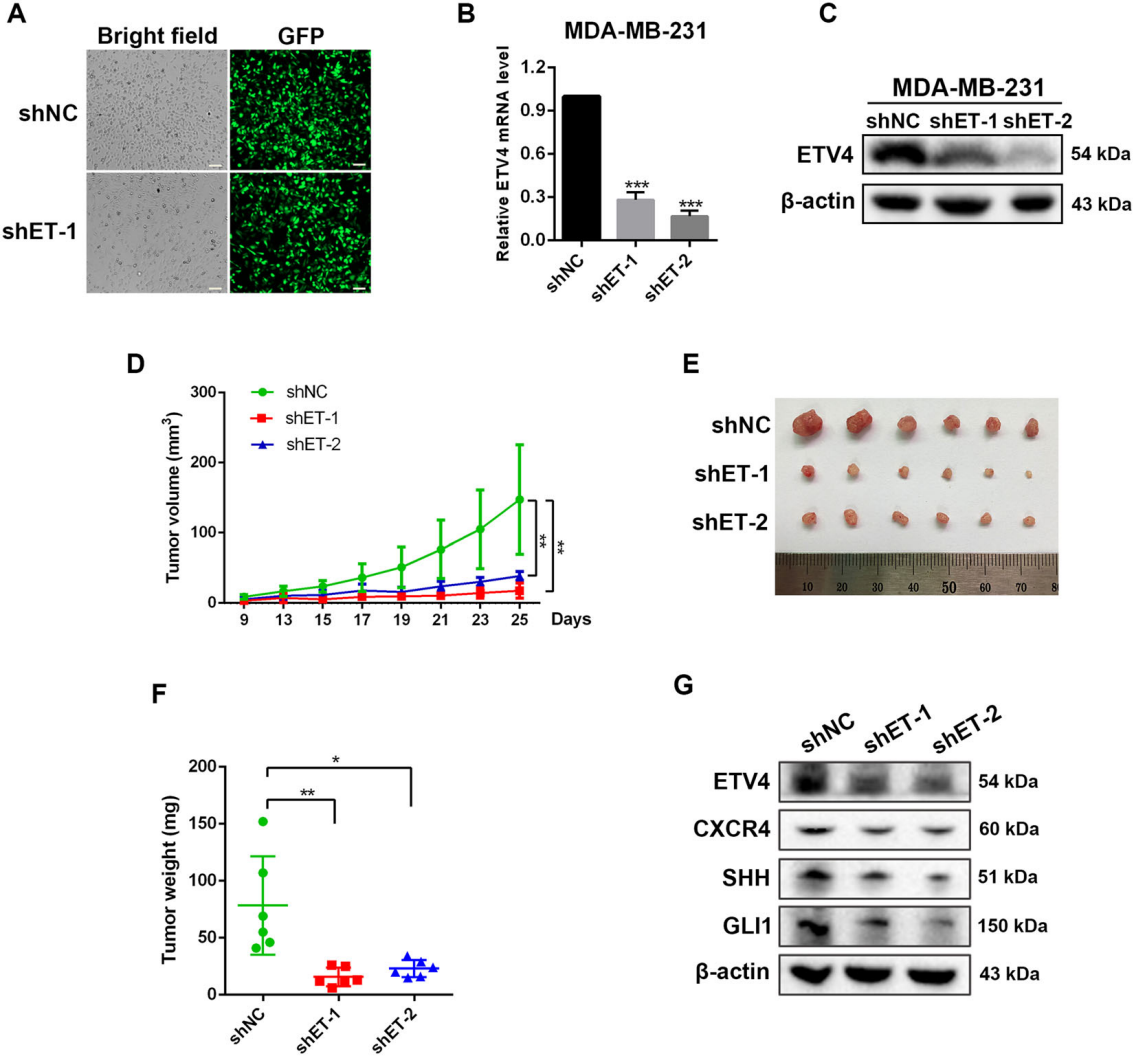
ETV4 loss retarded growth of breast tumor xenograft. **A** Efficient transduction of MDA-MB-231 cells by GFP-expressing lentiviral vector carrying short shRNAs for ETV4 as reflected by green fluorescence. **B**, **C** Establishment of stable ETV4 knockdown MDA-MB-231 cells as confirmed by quantitative RT‐PCR (**B**) and western blotting assay (**C**). **D**–**F** Nude mice (*n* = 6 each group) were subcutaneously implanted with control or shETV4 MDA-MB-231 cells. Tumor volumes were measured (**D**). Twenty-five days after inoculation, tumors were isolated (**E**) and weighted (**F**). **G** Immunoblots of ETV4, CXCR4, SHH and GLI1 in harvested MDA-MB-231 tumors. Data are presented as mean ± SD, *n* = 3. *P < 0.05, **P < 0.01, ***P < 0.001.

To probe the effect of ETV4 on tumor growth in vivo, we first established stable ETV4 knockdown MDA-MB-231 cells using lentiviral vectors. A high lentiviral infection efficiency was achieved as shown in Fig. 6A. After two weeks of screening with puromycin, cells with stable knockdown of ETV4 were established, as evidenced by significantly lower expression of ETV4 in the shETV4 groups (Fig. 6B, C). We then subcutaneously injected shETV4 cells into nude mice, ETV4 knockdown strongly retarded tumor growth and much smaller tumors were observed in mice inoculated with shETV4 cells compared with mice inoculated with control cells (Fig. 6D–F), which is consistent with previous reports^52,53^, and indicates that ETV4 is important for in vivo tumor growth. In isolated tumors, the protein expression levels of CXCR4 (Fig. 6G, Supplementary Fig. 5), SHH and GLI1 were decreased in shETV4 groups (Fig. 6G), which is consistent with our in vitro assay-based finding that ETV4 activates SHH signaling pathway by positively regulating CXCR4.

## Discussion

**Fig. 7:**
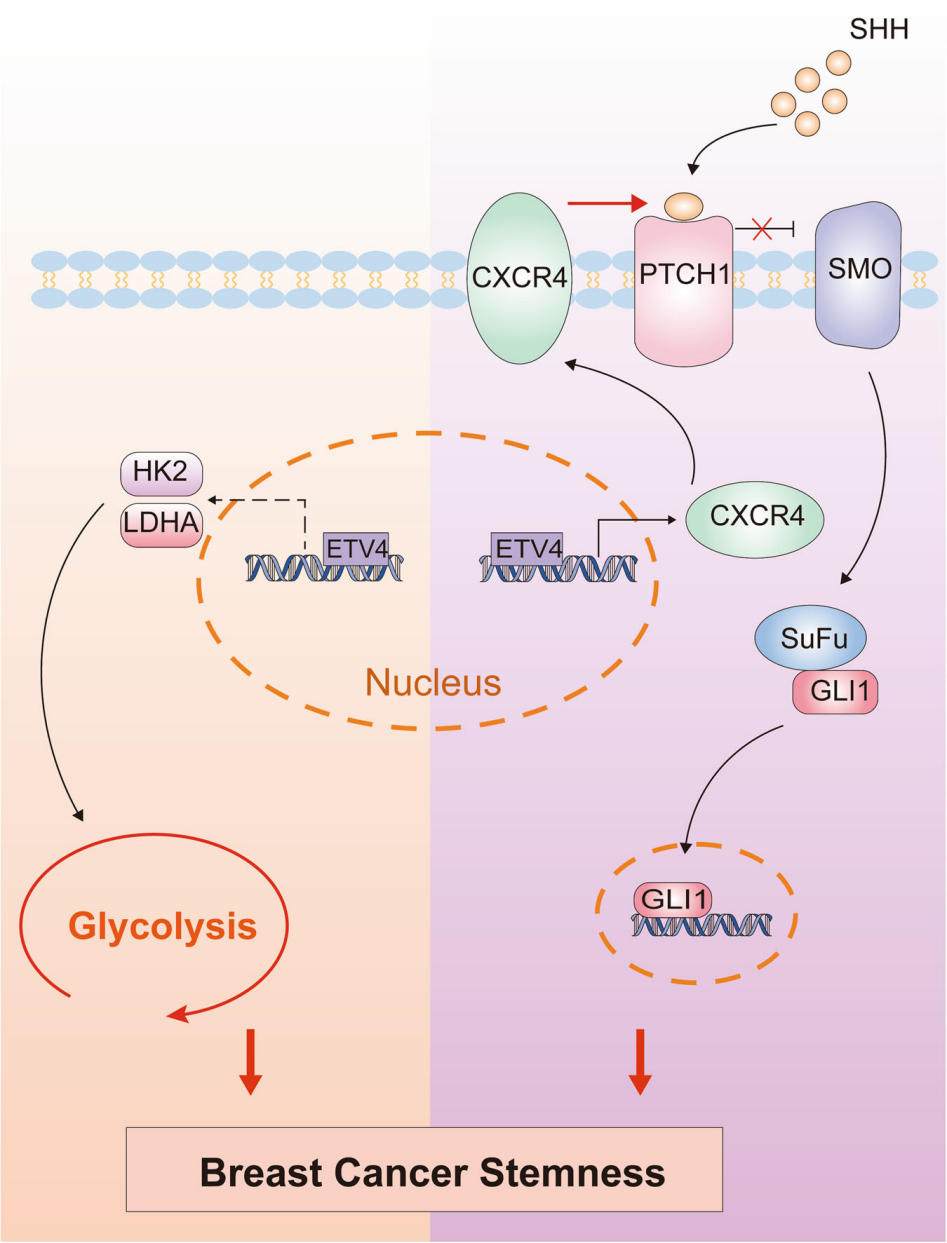
Schematic of the mechanism of action of ETV4 in promoting breast cancer stemness. ETV4 promotes BCSC maintenance, on the one hand, by promoting transcription of HK2 and LDHA to augment glycolytic flux, and onthe other, by transcriptionally activating CXCR4 expression to stimulate SHH/GLI1 signaling cascade.

The ^18^fluorodeoxyglucose positron-emission tomography widely used in clinical practice has shown that most human cancers depend on a glycolytic phenotype. Advantages conferred to tumor cells by this glycolytic metabolism, such as supporting macromolecular synthesis and surviving in hypoxic conditions, have led to the consensus that targeting glycolysis is a promising way for therapeutic intervention against cancer^54^. CSCs are closely correlated with tumor initiation, metastasis and treatment failure. In contrast to differentiated cancer cells, CSCs are metabolically plastic and rely on either glycolysis or OXPHOS^55–57^. In this study, we reveal a previously unappreciated role for ETV4 in regulating glycolytic metabolism by promoting expression of glycolytic enzymes including HK2, LDHA. We demonstrate that ETV4 is required for maintenance of BCSCs that are glycolysis-dependent. Moreover, besides its involvement in breast cancer cell stemness by modulating glycolytic gene expression, we show that ETV4 also activates SHH signaling to promote breast cancer stemness via transcriptionally regulating its target gene CXCR4. These data collectively illustrate the role of ETV4 in facilitating breast cancer stemness through two distinct mechanisms (Fig. 7).

More than ten metabolic enzymes are involved in the glycolysis progress, many of which are co-upregulated in various types of cancer. A recent study has shown that the expression levels of GLUT1, HK1, PKM2, and LDHA are significantly higher in primary colorectal cancer and liver metastasis tissues than in normal mucosa^58^. Lee et al. reported that HK2, PFKL, ALDOA and PKM2 expression are increased in HCC tissues and correlates with a progression of cirrhosis to HCC^59^. These studies suggest that a collaboration between glycolytic enzymes is crucial for tumor progression. Hence, targeting key regulatory factors upstream of these glycolytic enzymes is an efficient and preferable way for therapy of cancer, as compared with targeting a single enzyme. Transcription factors have been identified as key players in orchestrating glycolytic gene expression, including c-MYC, HIF-1 and p53^14^. A more recent study by Li et al. demonstrated that SIX1 is a critical transcription factor involved in aerobic glycolysis by binding promoters to promote transcription of multiple glycolytic genes, such as *PFKL*, *ALDOA*, *PGK1*, *ENO1*, *PKM2*, and *LDHA*^25^. Here, we show that ETV4 is a novel transcription factor capable of regulating the expression of many glycolytic enzymes. ETV4 depletion led to significantly downregulated mRNA expression of glycolytic proteins including SLC2A1, ALDOA, GAPDH, PGK1, PGM1, ENO1, ENO2, PDK1 and LDHA in HCC and breast cancer cells, although its effect on the expression of HK2, which catalyzes the first step in glucose metabolism by phosphorylating glucose to produce glucose-6-phosphate, differed between HCC and breast cancer cells, with a reduced HK2 mRNA level observed only in ETV4-silenced breast cancer cells. Significantly, ETV4 expression in human breast cancer and HCC samples correlates with glycolytic signaling, highlighting a possible role of ETV4 in mediating glycolytic shift in human cancers. The idea that ETV4 promotes glycolysis activity is further confirmed by the finding that knockdown of ETV4 suppressed glucose uptake and lactate production in human breast cancer cell lines. In spite of an increasing understanding of the role of ETV4 in tumor progression, the current study, to our knowledge, provides the first evidence for involvement of ETV4 in cancer metabolism reprogramming. Considering the upregulated expression of ETV4 in breast cancer, targeting ETV4 may be an alternative strategy to inhibit glycolysis in breast cancer. And yet, it is necessary to determine whether ETV4 directly binds promotors to activate transcription of multiple glycolytic genes in further studies.

Accumulating evidence shows that BCSCs exhibit a glycolytic phenotype^44,60^. Peng et al. demonstrated that highly expressed PDK1, which prevents pyruvate from being metabolized by oxidative decarboxylation, is required for maintenance of BCSCs^60^. Our study shows that PDK1 expression is regulated by ETV4. By analyzing the transcriptional data of the CSC and non-CSC subpopulations in the human breast cancer cell line SUM149 (GSE132083), we find elevated expression of majority of glycolytic enzymes and ETV4 in the CSC group. In addition, we confirm that HK2 and LDHA expression is upregulated in BCSC-enriched spheres, and block of glycolysis using 2-DG strikingly inhibits mammosphere formation. These results are consistent with previous work and underscore that glycolytic metabolism is important for BCSCs. Since we have also shown that ETV4 knockdown inhibits glycolytic gene expression, it is rational that ETV4 plays a role in maintaining breast cancer stemness. Our study indicates that ETV4 expression is increased in the BCSC subpopulation and breast cancer spheres. Knockdown or overexpression of ETV4 significantly restrains or fosters breast cancer cell stem-like traits, and ETV4 loss suppresses the expression of stemness markers including c-MYC, OCT4, NANOG, LIN28, suggesting that ETV4 acts as a key regulator in BCSC maintenance. We propose that ETV4 promotes breast cancer stemness probably by altering glycolysis metabolism, as the increase in sphere formation capacity caused by ETV4 overexpression is reversed in the presence of a glycolysis inhibitor. Interestingly, we find that the other two members of the PEA3 family, ETV1 and ETV5, are also upregulated in the BCSC subpopulation. Besides, ETV1 expression is increased in MDA-MB-231 spheres and ETV5 expression upregulated in MCF7 spheres. It remains to be elucidated in further studies whether the three PEA3 subfamily members cooperate with each other to sustain stem-like characteristics in cancer cells.

Notch, Hedgehog, and Wnt pathways are highly conserved signaling pathways implicated in controlling development and tissue homeostasis, their persistent activation in CSCs plays a critical role in cancer stemness^45,61,62^. Our study reveals that ETV4 can activate SHH signaling to enhance breast cancer cell stemness. In addition, transcription of Notch1 and Notch4 is activated by ETV4 in breast cancer cells, suggesting the involvement of ETV4 in stimulating Notch signaling^63^. These results indicate that ETV4 can influence breast cancer stemness in ways other than regulating glycolysis. CXCR4 is known to regulate the stem cell populations in many tumors including breast cancer^64,65^. Here, we demonstrate that CXCR4 is a regulator of SHH signaling pathway and that modulation of SHH pathway activity by ETV4 is mediated through CXCR4, these findings highlight a significant role of the ETV4/CXCR4/SHH axis in BCSC maintenance. Notably, the tumor suppressor Capicua (CIC), a well-established transcriptional repressor of ETV4^66,67^, has recently been proved as an inhibitor of stem-like properties in breast cancer cells^68^, our study further reveals the molecular mechanism underlying it.

In summary, our study shows that ETV4 is a probable key transcription factor in metabolism rewiring by regulating expression of multiple glycolytic enzymes. Moreover, we demonstrate that ETV4 promotes BCSC maintenance by, on the one hand, regulating glycolysis activity, and on the other, stimulating SHH signaling cascade that is mediated by CXCR4. Targeting ETV4 represents a potential therapeutic approach to disrupt cell metabolism and tumor stemness in the treatment of breast cancer.

## Materials and methods

### Cell culture and human samples

Human breast cancer cell lines MDA-MB-231 and MCF-7 were kindly provided by Dr. Zhang (Institute of clinical pharmacology, Central South University) and cultured in RPMI-1640 medium (Gibco) supplemented with 10% (v/v) fetal bovine serum (Gibco). Human hepatocellular carcinoma (HCC) cell lines HepG2 and Huh7 were gifts from Dr. Zhu (Institute of clinical pharmacology, Central South University), they were cultured in Dulbecco’s modified Eagle’s medium (Gibco) supplemented with 10% fetal bovine serum. All cell lines were maintained at 37 °C in a humidified incubator containing 5% CO_2_ and authenticated by short tandem repeat analysis before use. Freshly resected breast cancer and adjacent normal tissues were collected form patients undergoing surgical resection of breast cancer at Xiangya Hospital of Central South University. Tissue samples were immediately snap‐frozen and stored in liquid nitrogen after resection until further RNA extraction. This study was approved by the Ethics Committee of Xiangya Hospital and all patients provided written informed consent prior to sample collection.

### Transfection and generation of stable cell line

Transient transfection of siRNAs and plasmids into cells was achieved using the RNAiMAX reagent (Invitrogen) and Lipofectamine 3000 reagent (Invitrogen) respectively as per the manufacturer’s protocols. The siRNAs targeting ETV4 (#1: 5′-GGGCAGAGCAACGGAAUUU-3′, #2: 5′-GAAUGGAGUUCAAGCUCAU-3′), targeting CXCR4 (5′-CAAGCAAGGGTGTGAGTTT-3′) and control siRNA (5′-UUCUCCGAACGUGUCACGU-3′) were purchased from GenePharma (Shanghai, China). Human ETV4-expressing vector (EX-T8074-M02) and CXCR4-expressing vector (EX-A0419-M02) were purchased from GeneCopoeia (Guangzhou, China). To generate stable knockdown cell lines, lentiviral particles equipped with short hairpin RNAs (shRNAs) targeting ETV4 were purchased from GeneChem (Shanghai, China), cells infected with lentiviruses were treated with 2 μg/ml puromycin for two weeks to select shRNA-expressing cells.

### Western blot

Cells were lysed on ice for 30 min in RIPA lysis buffer supplemented with protease inhibitors and a phosphatase inhibitor cocktail (Bimake), followed by centrifugation at 12,000 g for 10 min. The protein concentration was measured using a BCA Assay Kit (Beyotime, Shanghai, China). Equal amounts of protein were resolved by SDS–polyacrylamide gel electrophoresis and transferred onto polyvinylidene difluoride membranes. After blocking, membranes were incubated with primary antibodies at 4 °C overnight and secondary antibodies at room temperature for 1 h. The immunoblots were visualized by ECL chemiluminescence (GE healthcare, Buckinghamshire, UK) using a Bio-Rad gel image analysis system. The following primary antibodies were used in this study: anti-ETV4 (sc-113, Santa Cruz), anti-HK2 (22029-1-AP, Proteintech), anti-LDHA (19987-1-AP, Proteintech), anti-PDK1 (3062 T, Cell Signaling Technology), anti-c-MYC (10828-1-AP, Proteintech), anti-OCT4 (11263-1-AP, Proteintech), anti-NANOG (14295-1-AP, Proteintech), anti-LIN28 (11724-1-AP, Ptoteintech), anti-SHH (ab53281, Abcam), anti-GLI1 (66905-1-Ig, Proteintech), anti-CXCR4 (60042-1-Ig, Proteintech), anti-β-actin (A1978, Sigma-Aldrich).

### Dual luciferase reporter assay

Cells seeded in 12-well plates with a confluence rate of ~50% were transfected with control siRNA or ETV4-targeting siRNA. Twenty-four hours later, experimental plasmids (pGL-3 basic, pGL-3 prom.HK2, pGL3-prom.LDHA) were transfected into cells and pRL-TK plasmid was simultaneously transfected for normalization. After culture for 24 hours, relative luciferase activity was measured using the Dual-Luciferase Reporter Assay System (Promega) according to the manufacturer’s instructions.

### Tumor xenograft study

Four-six weeks old female BALB/c nude mice purchased from Hunan SJA Laboratory Animal Co., Ltd were housed under specific pathogen-free conditions under a 12 h light/dark cycle. They were randomly assigned into three groups (shET-1, shET-2 and shNC). ETV4 stable knockdown MDA-MB-231 cells (1 × 10^7^) and their control cells were injected subcutaneously into the flank of the mouse in a volume of 100 μl medium. The tumor volumes were measured every three days using a digital caliper and calculated according to the formula: *V* = *a × b*^2^*/2*, where *a* represents the long diameter and *b* the short diameter of a tumor. After a 4-week period to monitor tumor growth, mice were sacrificed and the xenografted tumors were isolated for further analysis. All mouse studies were performed without blinding and approved by the Experimental Animal Ethics Committee of Central South University and conducted in compliance with established institutional guidelines.

### RNA extraction and quantitative RT–PCR

Total RNAs were extracted using RNAiso Plus (9108, TaKaRa) according to the manufacturer’s instructions. After complementary DNA was synthesized using the PrimeScript™ RT reagent Kit (RR047A, TaKaRa), real-time quantitative PCR was conducted on the Roche LightCycler 480 System using TB Green Premix Ex Taq II reagent (RR820A, TaKaRa). Relative mRNA expression was calculated using the 2^−ΔΔCt^ method and *ACTB* was used as an internal control. The primer sequences were listed in Supplementary Table 1.

### RNA-seq analysis

RNA sequencing of MDA-MB-231 cells transfected with siRNA for ETV4 or control siRNA was performed by Illumina Novaseq (Novogene, Beijing, China). After assessing RNA integrity using the Bioanalyzer 2100 system (Agilent Technologies, CA, USA), mRNA purification from total RNA was achieved using poly-T oligo-attached magnetic beads to construct RNA library, which was sequenced and 150 bp paired-end reads were generated. The sequencing data were aligned to human reference genome (hg 38) using Hisat2, and the read counts mapped to each gene were calculated by featureCounts. Differential gene expression analysis was carried out using the DESeq2 package.

### Gene set enrichment analysis

Gene set enrichment analysis (GSEA) to explore ETV4-associated molecular functions in breast cancer tissues was carried out by using TCGA RNA-seq data of 1104 breast cancer samples downloaded from UCSC Xena (https://xena.ucsc.edu/). The patients were ranked according to *ETV4* mRNA expression and divided into 2 groups based on the median *ETV4* expression. Pre-defined gene sets were acquired from the Molecular Signatures Database (MSigDB) and published studies^42,69^. Gene sets with a false discovery rate (FDR) less than 0.25 were considered significant, as previously reported^69,70^.

### Sphere formation assay

Cells were seeded in 6-well ultra-low attachment plates (Corning) at a concentration of 1 × 10^4^ cells/well and cultured for 10 days in serum-free DMEM-F12 (Gibco) supplemented with 20 μL/mL B27 (Gibco), 20 ng/mL epidermal growth factor (Peprotech), and 20 ng/mL basic fibroblast growth factor (Peprotech). Mammospheres with diameter >100 μm were counted.

### Flow cytometry analysis

The proportion of cancer stem cells was measured on a Cytek Athena flow cytometer by dual-staining for CD44 and CD24 using anti-CD44-APC (103011, BioLegend) and anti-CD24-PE antibodies (311105, BioLegend) according to the manufacturer’s instructions. Briefly, trypsinized cells were centrifugated and resuspended, followed by addition of 1.25 μL of anti-CD44-APC and 5 μL of anti-CD24-PE per 100 μL single cell suspension. After an incubation on ice for 20 min in the dark, cell samples were washed and subject to flow cytometry analysis. The subpopulation of CD44^+^/CD24^−^ cells were analyzed using the FlowJo software.

### Measurement of glucose uptake and lactate production

Twenty-four hours after siRNA transfection, cells were incubated with fresh medium for 48 h and the medium was then collected. Glucose and lactate levels in the medium were measured by an automatic biochemical analyzer (AU680, Beckman Coulter, USA) at the Biochemical Laboratory of Xiangya Hospital (Changsha, China). The relative glucose uptake rate and lactate production rate were normalized to cell number.

### Immunohistochemistry

Tumors resected from mice were formalin-fixed and paraffin-embedded before cutting into 4 μm thick sections, which were then baked at 60 °C for 30 minutes, dewaxed with xylene and hydrated with gradient ethanol. After antigen retrieval and blocking of nonspecific staining, tumor sections were incubated with a primary antibody against CXCR4 (60042-1-Ig, Proteintech) at 4 °C overnight, followed by incubation with a secondary antibody at room temperature for 2 hours. The stained sections were viewed and photographed with a Leica inverted microscope after visualizing with DAB.

### Statistical analysis

Data analyses were performed using GraphPad Prism or R. Results were presented as mean ± standard deviation (SD). Two-tailed Student’s *t* test was used to compare two groups for statistical differences. The Kaplan-Meier method and log-rank test were utilized to estimate distant relapse free survival and compare survival differences. Normalized gene expression data were z score transformed for optimal heatmap presentation. Sample sizes were chosen based on previous literature. Differences were considered statistically significant when *P* < 0.05 (*P < 0.05; **P < 0.01; and ***P < 0.001).

## Supplementary information

Supplementary Figure 1

Supplementary Figure 2

Supplementary Figure 3

Supplementary Figure 4

Supplementary Figure 5

Supplementary Table 1
