# Supplementary figures and images for: ETV4 promotes breast cancer cell stemness by activating glycolysis and CXCR4-mediated sonic Hedgehog signaling
Source: Cell Death Discov. 2021 May 29;7:126. doi: 10.1038/s41420-021-00508-x (PMC8164634; doi:10.1038/s41420-021-00508-x)

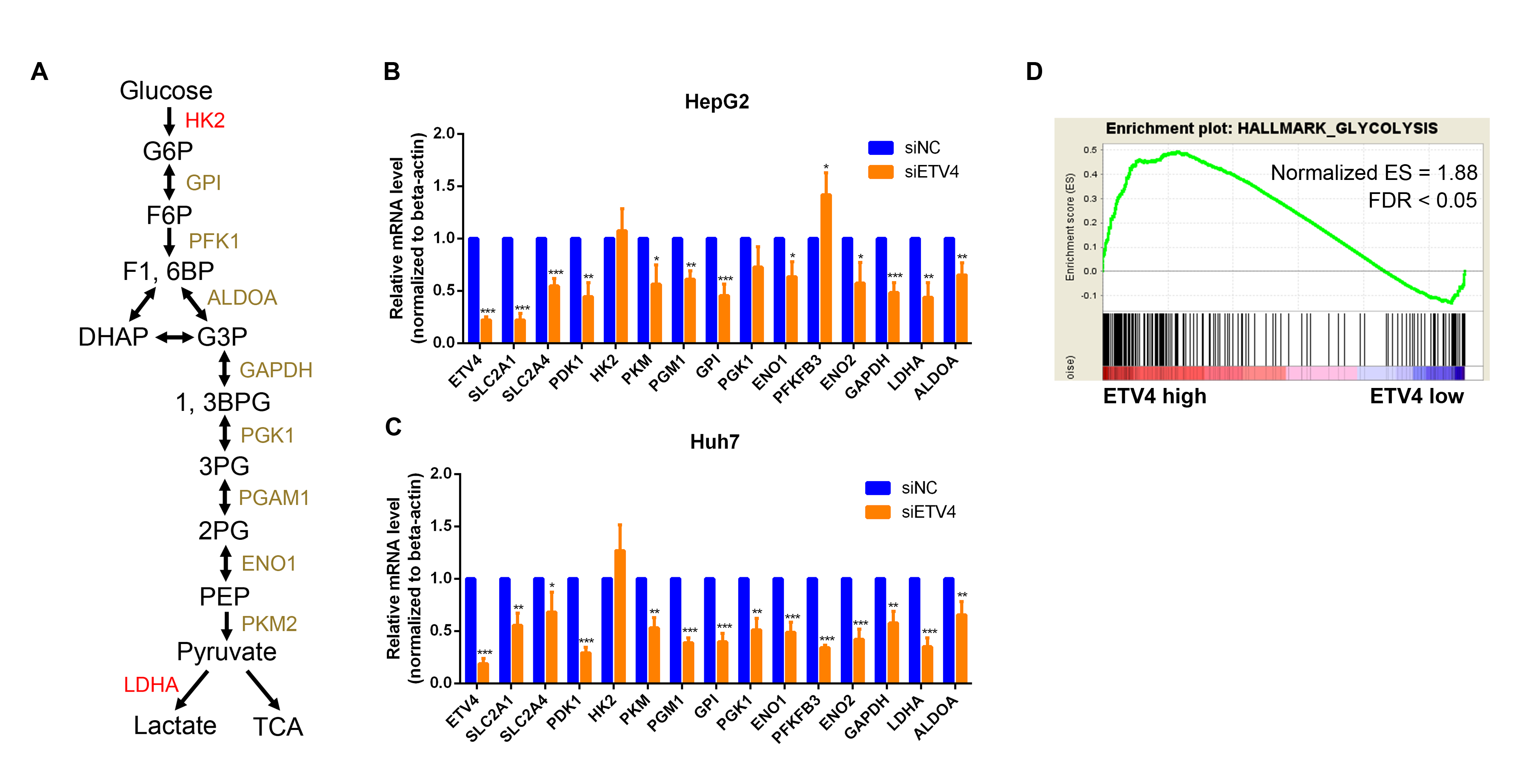

Supplement: Supplementary file 1 — Supplementary Figure 1 [file 41420_2021_508_MOESM1_ESM.tif]

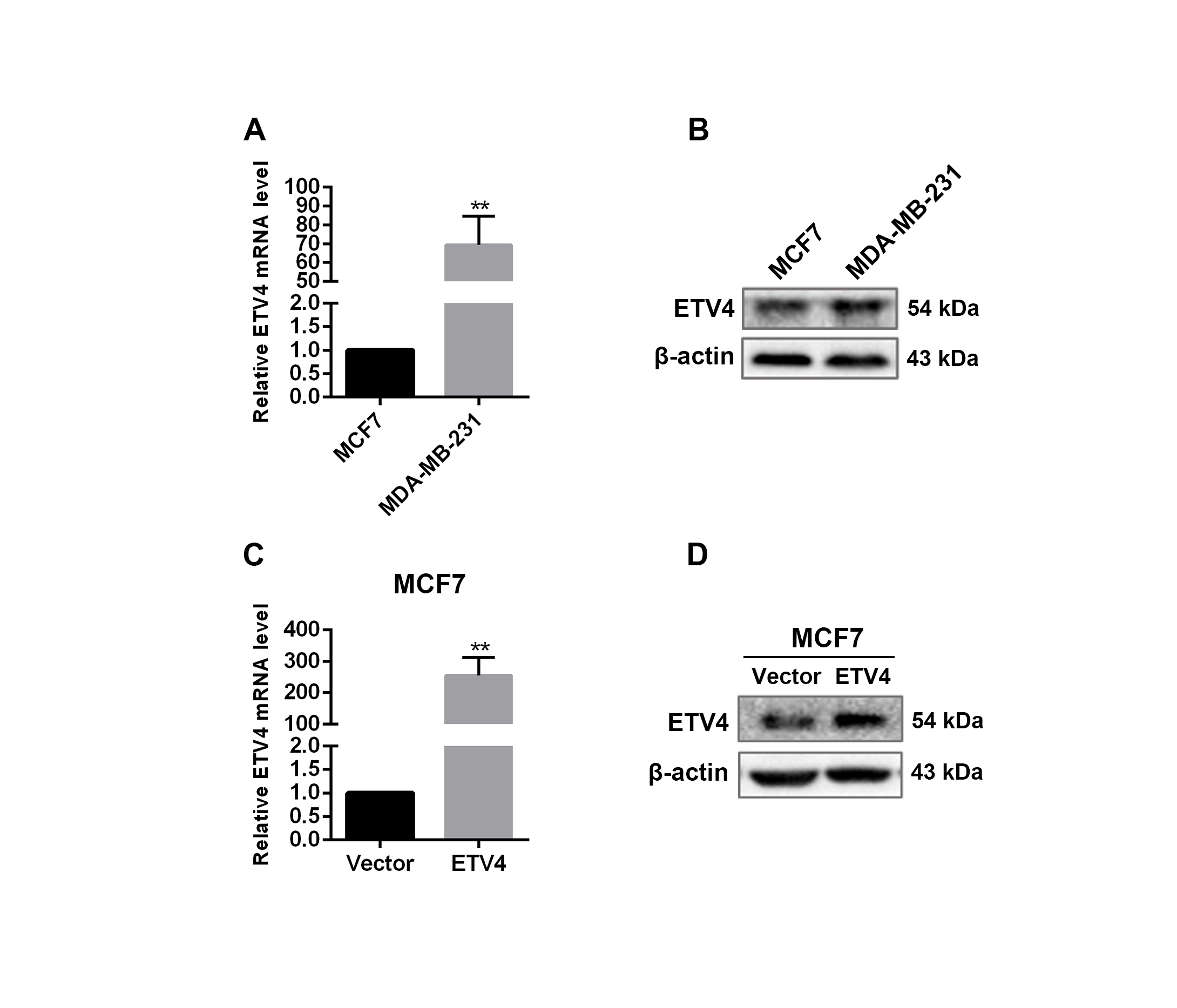

Supplement: Supplementary file 2 — Supplementary Figure 2 [file 41420_2021_508_MOESM2_ESM.tif]

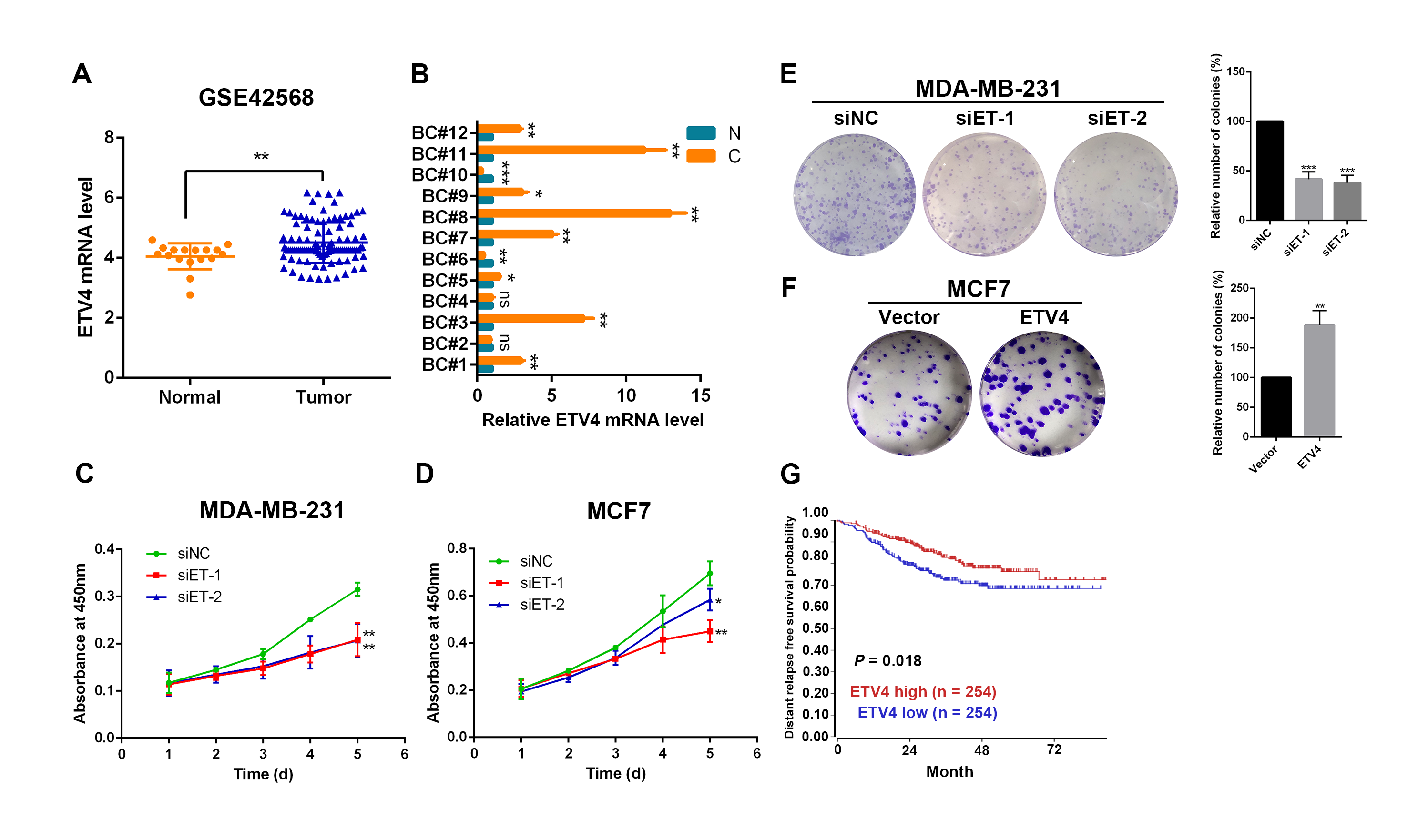

Supplement: Supplementary file 3 — Supplementary Figure 3 [file 41420_2021_508_MOESM3_ESM.tif]

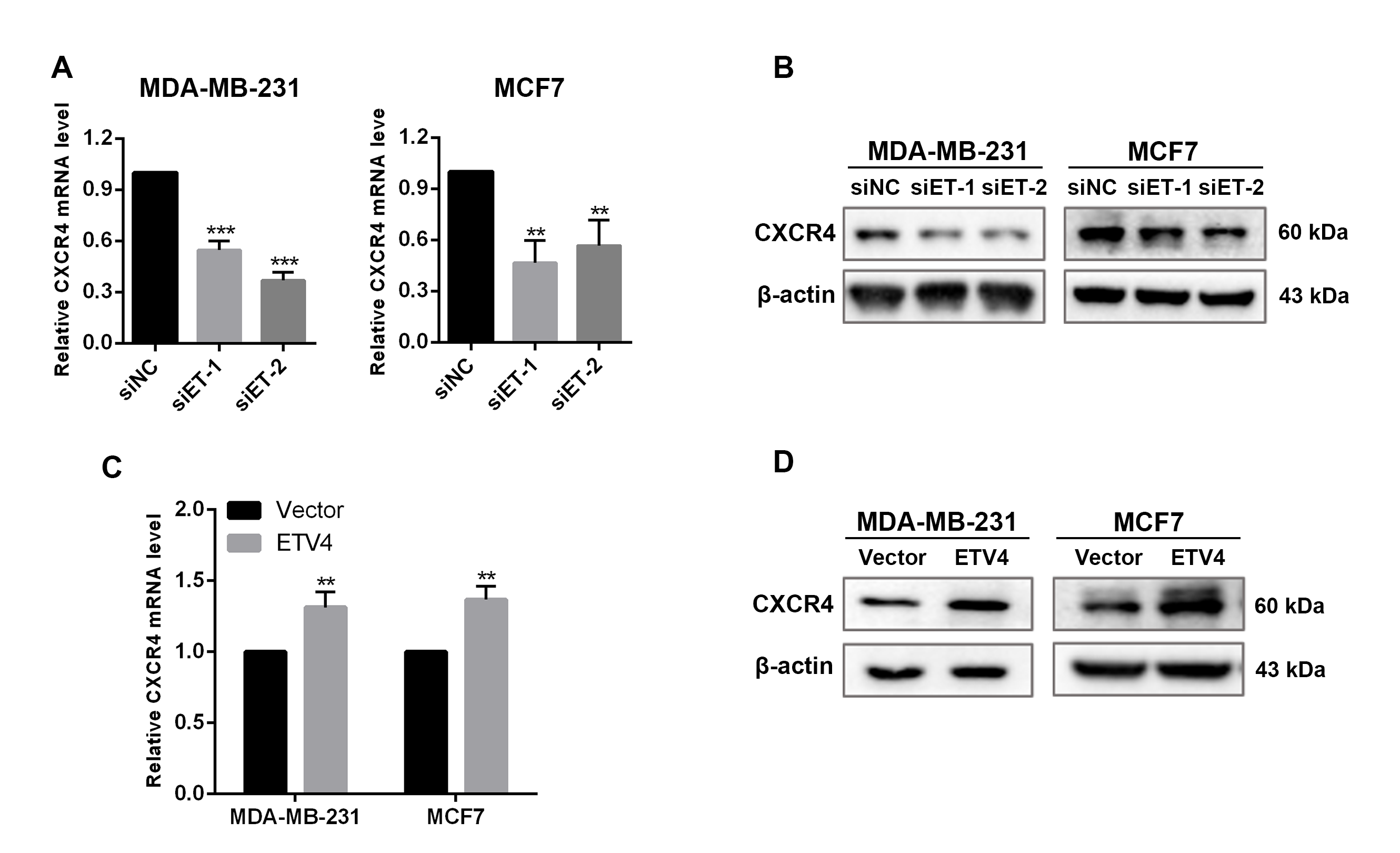

Supplement: Supplementary file 4 — Supplementary Figure 4 [file 41420_2021_508_MOESM4_ESM.tif]

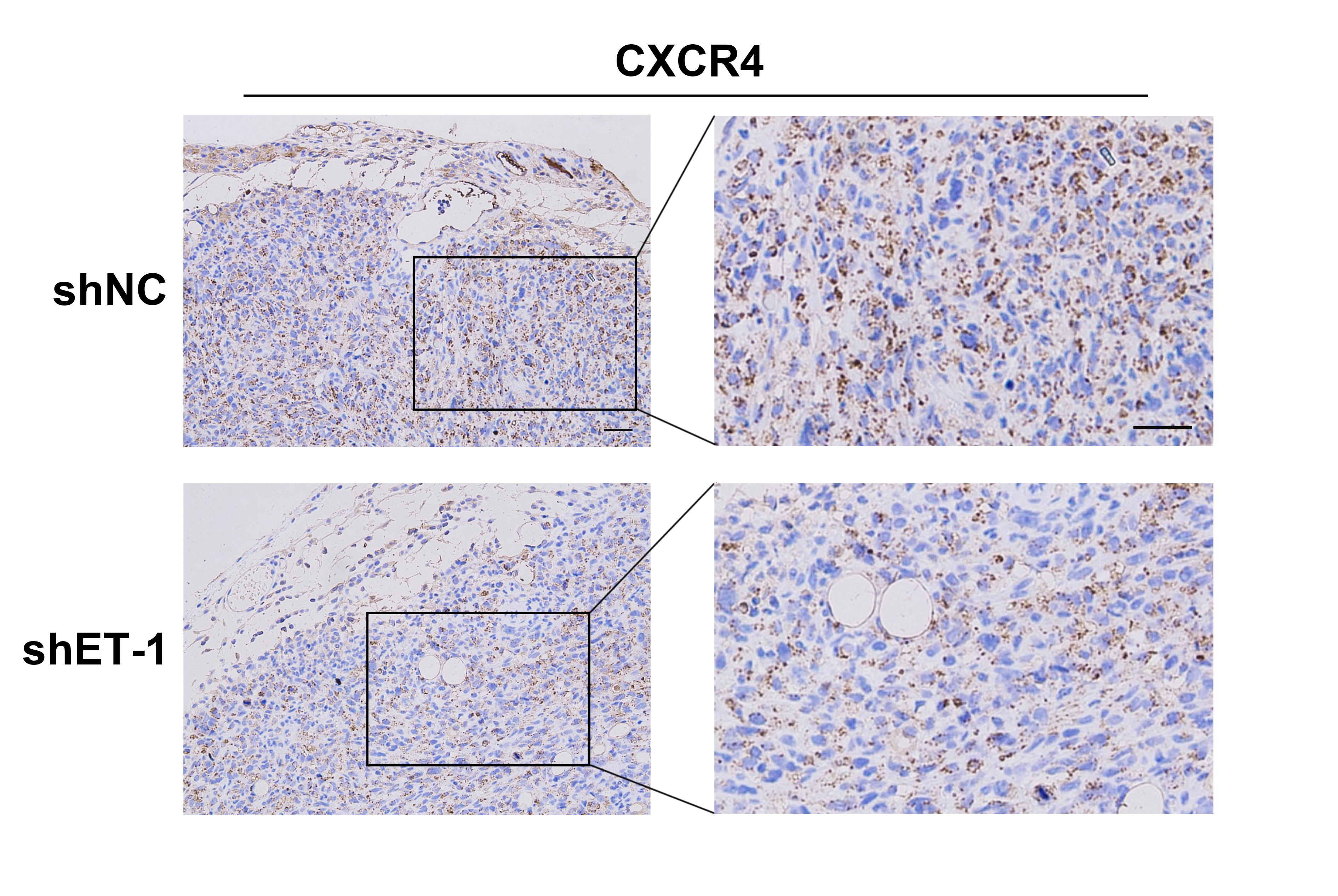

Supplement: Supplementary file 5 — Supplementary Figure 5 [file 41420_2021_508_MOESM5_ESM.tif]
